# Xanthohumol inhibits colorectal cancer cells via downregulation of Hexokinases II-mediated glycolysis

**DOI:** 10.7150/ijbs.37481

**Published:** 2019-09-07

**Authors:** Wenbin Liu, Wei Li, Haidan Liu, Xinfang Yu

**Affiliations:** 1 Department of Pathology, Hunan Cancer Hospital and The Affiliated Cancer Hospital of Xiangya School of Medicine, Central South University, Changsha, Hunan 410006, P.R. China; 2 Department of Radiology, The Third Xiangya Hospital of Central South University, Changsha, Hunan 410013, P.R. China; 3 Clinical Center for Gene Diagnosis and Therapy, The Second Xiangya Hospital of Central South University, Changsha, Hunan 410011, P.R. China; 4 Department of Cardiovascular Surgery, The Second Xiangya Hospital of Central South University, Changsha, Hunan 410011, P.R. China; 5 Department of Cancer Biology, Lerner Research Institute, Cleveland Clinic, 9500 Euclid Avenue, Cleveland, Ohio 44195, USA

**Keywords:** Xanthohumol, HK2, glycolysis, Akt, colorectal cancer

## Abstract

Deregulation of glycolysis is a common phenomenon in human colorectal cancer (CRC). In the present study, we reported that Hexokinase 2 (HK2) is overexpressed in human CRC tissues and cell lines, knockout of HK2 inhibited cell proliferation, colony formation, and xenograft tumor growth. We demonstrated that the natural compound, xanthohumol, has a profound anti-tumor effect on CRC via down-regulation of HK2 and glycolysis. Xanthohumol suppressed CRC cell growth both *in vitro* and *in vivo*. Treatment with xanthohumol promoted the release of cytochrome C and activated the intrinsic apoptosis pathway. Moreover, our results revealed that xanthohumol down-regulated the EGFR-Akt signaling, exogenous overexpression of constitutively activated Akt1 significantly impaired xanthohumol-induced glycolysis suppression and apoptosis induction. Our results suggest that targeting HK2 appears to be a new approach for clinical CRC prevention or treatment.

## Introduction

Colorectal cancer (CRC) is the third most common type of cancer worldwide and a leading cause of cancer-related death 1. Although surgery represents the mainstay of treatment in early cases, a large number of patients are diagnosed in an advanced stage, and sometimes also distant metastases are present. Chemotherapy and targeted therapy are, therefore needed. However, Drug resistance remains the major obstacle which influences response and leads to recurrent disease 2, 3. Discovery of new anti-tumor agents and identification of novel targets will improve survival and offer patients a greater likelihood of benefit for CRC clinical treatment.

Most cancer cells preferentially take glycolysis as their energy source even in the presence of oxygen, this phenomenon is often named “Warburg effect” 4. The first irreversible step of glycolysis phosphorylates glucose to glucose-6-phosphate, which is catalyzed by HKs. So far, four HK isozymes, including HK1, 2, 3, and 4, have been identified in mammals 5. HK1 and HK2 bind to the mitochondrial voltage dependent anion channel (VDAC) and play a crucial role in maintaining of mitochondria potential. Disruption of the binding of HK with VDAC causes mitochondrial dysfunction and induces apoptosis 6, 7. Exception from other hexokinase family members, HK2 is always overexpressed in human cancers. Besides the acceleration of glucose uptake, highly expressed HK2 confers apoptosis resistance by form the HK2-VDAC complex in mitochondria of cancer cells 8, 9. Therefore, HK2, in particular, is regarded as a potential target for anticancer therapy.

Xanthohumol (2',4',4-trihydroxy-6'-methoxy-3'- prenylchalcone), a polyphenol chalcone from hops (Humulus lupulus), has received increasing attention due to its multiple pharmacological activities 10. Increasing evidence suggested the anticancer activity of xanthohumol against colorectal cancer 11, leukemia 12, non-small cell lung cancer 13, breast 14, and cervical 15 cancers. However, the underlying mechanisms remain unclear. In the present study, we investigated the anti-tumor effect of xanthohumol *in vitro* and *in vivo*. Our results demonstrated that xanthohumol suppressed CRC cells via suppression of HK2 and glycolysis. This study suggests that xanthohumol may serve as a new anti-tumor agent which deserves further study.

## Materials and Methods

### Cell culture and reagents

FHC, CCD841 CoN, HT29, SW480, LOVO, HCT116, and SW620 were purchased from American Type Culture Collection (ATCC, Manassas, VA). The cells were cultured at 37°C in a humidified incubator with 5% CO2 according to ATCC protocols. All cell lines were routinely checked for mycoplasma contamination and subjected to cytogenetically test to confirm identity. Cell culture media, FBS, and supplements were from Invitrogen (Grand Island, NY).

The compounds used in this study, including diosmetin, nobiletin, osthole, sinomenine, xanthohumol, isoliquiritigenin, oxymatrine, bergenin, bButein, sophocarpin, curcumol, sesamin, phlorizin, gastrodin, limonin, and paeonol were purchased from Selleck Chemicals (Houston, TX). SDS, Tris, and NaCl for molecular biology and buffer preparation were purchased from Sigma (St. Louis, MO). Lipofectamine 2000 was purchased from Invitrogen (#11668019, Carlsbad, CA).

Antibodies against HK2 (#2867, IB: 1:2000, IHC:1:200), HK1 (#2024, IB: 1:2000), p-EGFR (#3777, IB: 1:1000), p-Akt (#4060, IB: 1:1000, IHC: 1:100), p-ERK1/2 (#4370, IB: 1:1000), α-Tubulin (#2144, IB:1:10000), Bax (#14796, IB: 1:1000), Cytochrome c (#4280, IB: 1:1000), VDAC1 (#4866, IB:1:2000), cleaved-caspase 3 (#9664, IB: 1:2000) and cleaved-PARP (#5625, IB: 1:2000) were obtained from Cell Signaling Technology, Inc. (Beverly, MA). Antibodies against β-actin (A5316, IB: 1:10000) was from Sigma-Aldrich (St. Louis, MO). Antibodies against Ki67 (ab16667, IHC: 1: 250), donkey anti-rabbit IgG H&L (Alexa Fluor® 568) (ab175470), and donkey anti-rabbit IgG H&L (Alexa Fluor® 488) (ab150073) were purchased from Abcam (Cambridge, UK). Secondary antibodies, including anti-rabbit IgG HRP (#7074) and anti-mouse IgG HRP (#7076), were purchased from Cell Signaling Technology. Antibody conjugates were visualized by chemiluminescence (ECL; cat#34076, Thermo Fisher).

### MTS assays

Cell viability was measured using a CellTiter 96® AQueous One Solution Cell Proliferation Assay kit (MTS) purchased from Promega Corp. (#G3580, Madison, WI). It was used according to the manufacturer's protocol. Briefly, cells (3000 per well) were seeded into a 96-well plate. After 24 h, cells were treated with or without different concentrations of xanthohumol and incubated for various time points. Cell viability was quantified by MTS assay. Three independent experiments were performed.

### Plate colony-formation assay

The cells were treated with xanthohumol or control for 24 h and seeded in a 6-cm plate (300/well). The cultures were maintained for 2 weeks at 37°C in a 5% CO_2_ incubator. The colonies were fixed with 4% paraformaldehyde, stained with 0.5% crystal violet, and counted under a microscope. Three independent experiments were performed as indicated.

### Immunofluorescence

Cells were seeded in chamber slides and fixed in 4% paraformaldehyde and permeabilized in 0.5% Triton X-100 for 20 min, followed by blocking in 5% BSA for 1 h and incubated with Histone H3 pS10 or cleaved-caspase 3 antibodies at 4°C in a humidified chamber overnight. Alexa Fluor 488 dye-labeled anti-rabbit IgG or Alexa Fluor 568 dye-labeled anti-rabbit IgG were used as secondary antibodies. Nuclei were counterstained with DAPI (P36935, Thermo Fisher Scientific). Samples were viewed using the confocal fluorescence microscope system (Nikon C1si; NIKON Instruments Co.).

### Western blotting

Protein samples were prepared with RIPA buffer (10 mM Tris-Cl (pH 8.0), 1 mM EDTA, 0.5 mM EGTA, 1% Triton X-100, 0.1% sodium deoxycholate, 0.1% SDS, 140 mM NaCl). Protein samples (20 μg) were separated by 10% SDS-PAGE, transferred onto PVDF membranes, and blocking with 5% non-fat milk. After overnight incubation with primary antibodies at 4°C, membranes were washed with TBST three times and then incubated with secondary antibodies at room temperature for 1 h. Immunobloting bands were visualized by an enhanced Western lightening plus-ECL kit (#32132, PIERCE, Rockford, IL). β-actin served as a loading control.

### Glucose uptake and lactate production measurement

Colorectal cancer cells were seeded in 10-cm plates and treated with different doses of xanthohumol for 24 h. Cells were seeded in 6-well plates (1×10^6^), after incubation for 5 h, the medium was discarded, and cells were incubated in fresh medium for another 6 h. Glucose and lactate levels were measured at the Laboratory of Hunan Cancer Hospital (Changsha, China). The relative glucose consumption rate and lactate production rate were normalized by the protein concentration of samples.

### Xenograft mouse model

All the experimentation for animals was approved by the Animal Ethics Committee of Hunan Cancer Hospital. Colorectal cancer cells (5× 10^5^) in 100 μL 1640 medium were inoculated s.c. into the right flank of 7-week-old female athymic nude mice. Tumor volume was determined by Vernier caliper every two days. For xanthohumol treatment, mice were given an i.p. injection of xanthohumol at a dose of 10 mg/kg every two days when tumor volume reached around 100 mm^3^. The control mice were administered vehicle. The body weight of each mouse was recorded, and tumor volume was determined by Vernier caliper every two days. Tumor volume was calculated following the formula of A × B^2^ × 0.5, wherein A is the longest diameter of tumor, B is the shortest diameter, and B^2^ is B squared. At the endpoint, the mice were sacrificed, and solid tumors were removed, weight, and photographed.

### Immunohistochemical analysis of tumor tissue

A human colorectal tissue array (BC05118e) was purchased from US Biomax, Inc. (Rockville, MD) and included 50 cases of adenocarcinoma and matched adjacent tissue. A Vectastain Elite ABC Kit (Vector Laboratories; Burlingame, CA) was used for immunohistochemical staining following the protocol. Briefly, after deparaffinized, and rehydrated, the slide was unmasked by submersion into boiling sodium citrate buffer (10 mM, pH 6.0) for 10 min, and then treated with 3% H_2_O_2_ for 10 min. 50% goat serum albumin in 1×PBS was used for blocking. The slides were incubated with the primary antibody at the cold room in a humidified chamber overnight. After washed and hybridized with the secondary antibody for 1 h at room temperature, the slides were stained using the Vectastain Elite ABC kit. The intensity was estimated using Image-Pro PLUS (v.6) and Image J (NIH) software programs. Statistical analyses were performed using Prism 5.0.

### Statistical analysis

Standard statistical methods were performed using Statistics Package for Social Science (SPSS) software (version 13.0; SPSS, Chicago, IL, USA). All data are presented as mean values ± S.D. as indicated and analyzed using the Student's *t*-test or ANOVA. A *p* value < 0.05 was considered statistically significant.

## Results

### HK2 is highly expressed in human colorectal cancer cells

**Figure 1:**
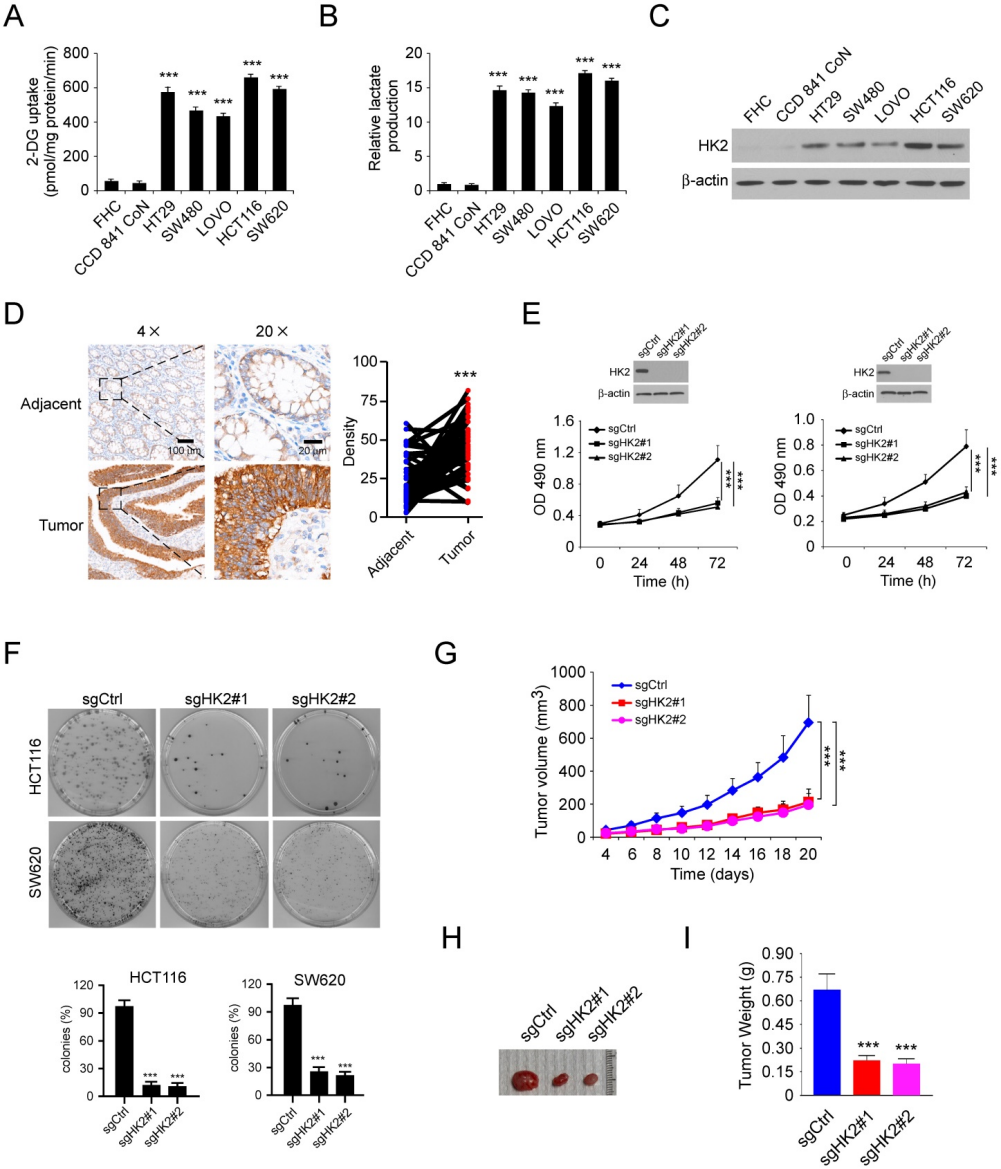
HK2 is required for the maintaining of tumorigenic properties in human colorectal cancer cells. A and B, 2-DG uptake (A) and normalized lactate production (B) in colorectal cancer cells and normal colon epithelial cells. The lactate production was normalized to the level of FHC cells. C, immunoblot (IB) analysis of HK2 expression in colorectal cancer cells and normal colon epithelial cells. D, immunohistochemistry (IHC) analysis of HK2 in colorectal cancer tissues and matched adjacent tissues. E, MTS analysis of cell proliferation of HK2 knockout and control HCT116 (left) and SW620 (right) stable cells. F, colony formation of HK2 knockout and control HCT116 (top) and SW620 (bottom) stable cells. G-I, average tumor volume (G), photographed xenograft tumors (H), and average tumor weight (I) of HCT116 sgCtrl and HCT116 sgHK2 xenografts. Data are shown as mean values ± S.D. ***p<0.001, a significant difference between groups as indicated.

We first investigated the glycolysis characteristics of a panel of human colorectal cancer (CRC) cells and two immortalized normal colon epithelial cells cultured under normoxic conditions. The results showed that the glucose consumption (Figure 1A) and lactate production (Figure 1B) were significantly upregulated in colorectal cancer cells, suggesting that CRC cells show an increased rate of aerobic glycolysis compared with normal cells. As the first rate-limiting enzyme of glycolysis, HK2 is expressed at high level in human cancer cells. We therefore tested the protein level of HK2 in CRC cells and immortalized normal colon epithelial cells. As result shown in Figure 1C, HK2 was markedly upregulated in CRC cells. The immunohistochemical (IHC) staining result showed that the expression of HK2 is significantly increased in the CRC tissues as compared with the paired adjacent tissues (Figure 1D). To assess the effect of HK2 on the proliferation of CRC cells, we generated HK2 stable knockout HCT116 (Figure 1E, left) and SW620 (Figure 1E, right) cell lines and validated sgRNAs that effectively depleted HK2 expression after transfection. Knockout of HK2 suppressed both anchorage-dependent cell growth (Figure 1E) and colony formation (Figure 1F). We further conduct the athymic nude mouse model to determine the role of HK2 in the tumorigenesis of CRC *in vivo*. The result showed that knockout of HK2 significantly inhibited tumor growth in HCT116 (Figure 1G-I) and HT29 xenograft mouse models (Figure S1A-C). These results indicate that HK2 may play a critical role in CRC tumorigenesis, and knockout of HK2 reduces tumorigenic properties.

### Xanthohumol inhibits CRC cells growth

**Figure 2:**
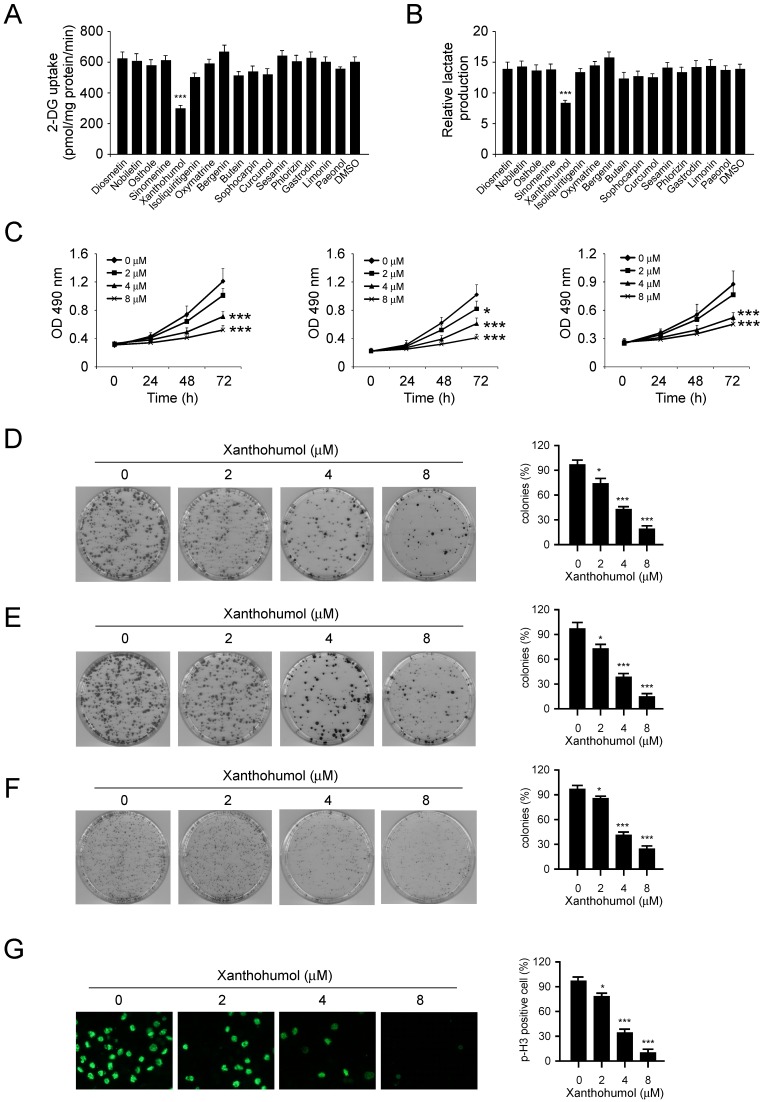
Inhibitory effects of xanthohumol on colorectal cancer cells. A and B, 2-DG uptake (A) and normalized lactate production (B) in colorectal cancer cells treated with different compounds. C, xanthohumol inhibits anchorage-dependent growth of HCT116 (left), HT29 (middle), and SW620 (right) cells. D-F, the effect of xanthohumol on colony formation of HCT116 (D), HT29 (E), and SW620 (F) cells. G, immunofluorescence analysis of Histone H3 S10 in HCT116 cells. Data are shown as mean values ± S.D. *p<0.05, ***p<0.001, a significant difference between groups as indicated.

In order to discover some natural compounds (Figure S2) that can specifically inhibit CRC cells via suppression of glycolysis, we first tested the glucose consumption and lactate production in HCT116 cells treated with various compounds. The results showed that only xanthohumol significantly inhibited glucose consumption (Figure 2A) and lactate production (Figure 2B) of HCT116 cells at the concentration of 5 μM. We therefore focused on xanthohumol for further study. As result showed in Figure 2C, xanthohumol inhibited cell proliferation in HCT116, SW620, and HT29 cells in a dose- and time-dependent manner. We then investigated the effects of xanthohumol on colony formation of these three CRC cells. The resulted showed that xanthohumol significantly decreased colony formation of CRC cells even at the concentration of 2 μM (Figure 2D-F). Because the phosphorylation of Histone H3 S10 is a marker for cell proliferation, we therefore examined the expression of Histone H3 S10 (p-H3 S10) using immunofluorescence. The result indicated that xanthohumol inhibited p-H3 S10 in HCT116 cells (Figure 2G), which further confirmed that xanthohumol decreased CRC cells' proliferation dose-dependently.

### Xanthohumol suppressed glycolysis and induces apoptosis in CRC cells

**Figure 3:**
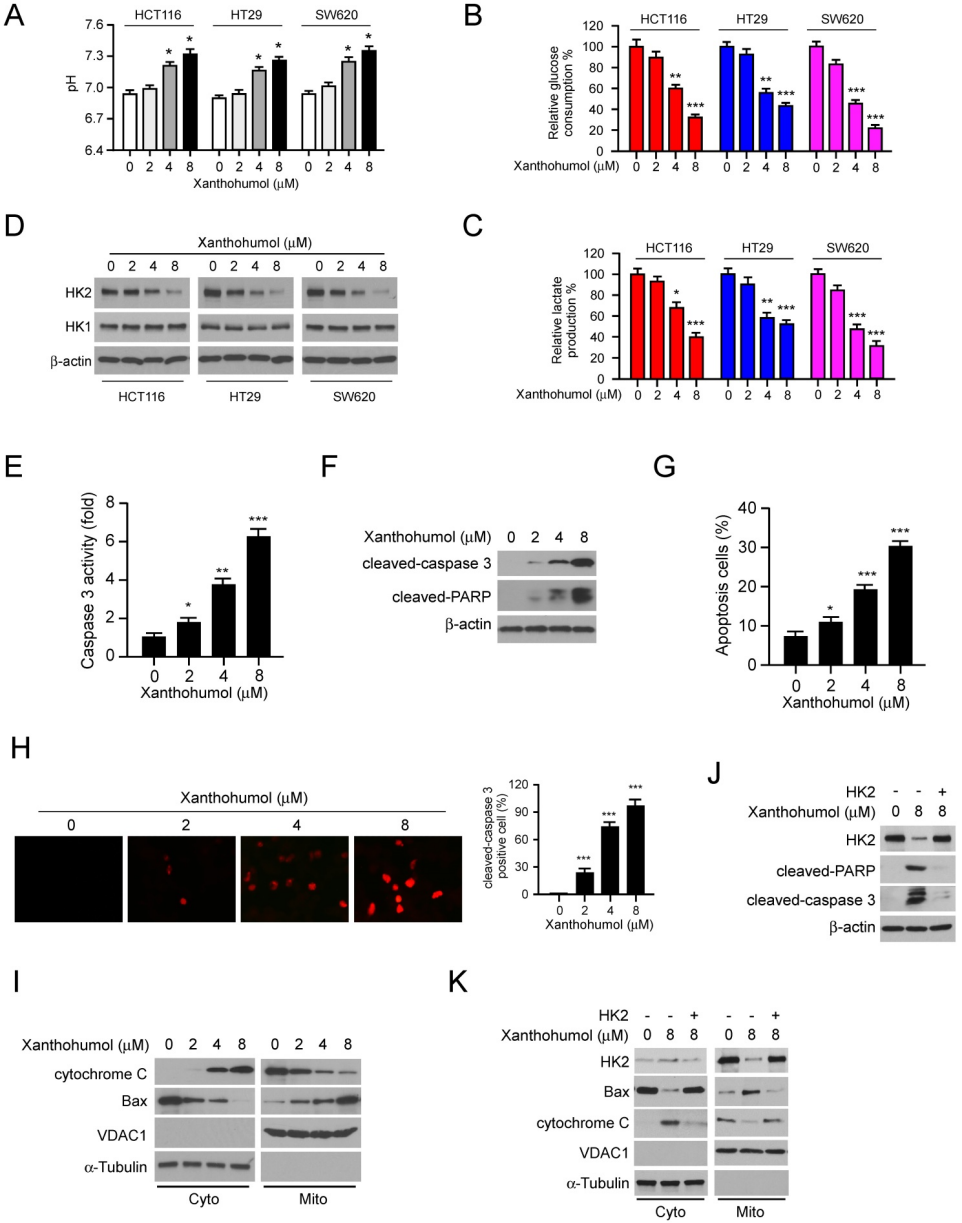
Xanthohumol suppresses glycolysis and induces apoptosis in colorectal cancer cells. A, pH value of cell culture medium form xanthohumol-treated colorectal cancer cells. B and C, glucose consumption (B) and lactate production (C) in xanthohumol-treated colorectal cancer cells. D, IB analysis of HK2 and HK1 expression in xanthohumol-treated colorectal cancer cells. E, normalized caspase 3 activity in xanthohumol-treated HCT116 cells. F and G, IB (F) and flow cytometry (G) analysis of cleaved-caspase 3 and -PARP in xanthohumol-treated HCT116 cells. H, immunofluorescence analysis of cleaved-caspase 3 in xanthohumol-treated HCT116 cells. I, IB analysis of cytochrome C and Bax expression in cytosolic and mitochondrial fractions of xanthohumol-treated HCT116 cells. Cyto, cytosolic fraction, Mito, mitochondrial fraction. J, overexpression of HK2 rescued xanthohumol-induced apoptosis in HCT116 cells. K, overexpression of HK2 rescued xanthohumol-induced mitochondrial apoptosis in HCT116 cells. Data are shown as mean values ± S.D. *p<0.05, **p<0.01, ***p<0.001, a significant difference between groups as indicated.

To further validate the inhibitory effect of xanthohumol on glycolysis, we analyzed the pH value in xanthohumol-treated CRC cell culture medium. The result showed that xanthohumol-treated CRC cells displayed a weaker capacity to decrease medium pH values than control cells (Figure 3A), suggesting that the efficacy of lactate production was impaired in xanthohumol-treated CRC cells. Consistently, glucose consumption (Figure 3B) and lactate production (Figure 3C) were significantly decreased in xanthohumol-treated CRC cells. Moreover, the immunoblotting data indicated that xanthohumol markedly decreased HK2, but not HK1protein level in a dose-dependent manner (Figure 3D). Localization of HK2 on mitochondrial confers apoptosis resistance in human cancer cells. Our data showed that xanthohumol significantly enhanced the activity of caspase 3 (Figure 3E), promoted the expression of cleaved-caspase 3 and -PARP (Figure 3F). The flow cytometry (Figure 3G) and immunofluorescence staining (Figure 3H) indicated that xanthohumol-induced apoptosis in HCT116 cells dose-dependently. Xanthohumol promoted the release of cytochrome C from mitochondria to the cytoplasm, and enhanced the mitochondrial localization of Bax (Figure 3I), indicating the activation of intrinsic apoptosis signaling. To determine whether downregulation of HK2 led to apoptosis in xanthohumol-treated cells, we overexpressed HK2 in HCT116 cells and further confirmed that transient transfection of HK2 rescued xanthohumol-induced apoptosis (Figure 3J) and attenuated the activation of mitochondrial apoptosis signaling (Figure 3K). These results suggest that xanthohumol suppressed the expression of HK2 and induced mitochondrial-associated apoptosis in CRC cells.

### Akt suppression is required for xanthohumol- mediated glycolysis inhibition in CRC cells

**Figure 4:**
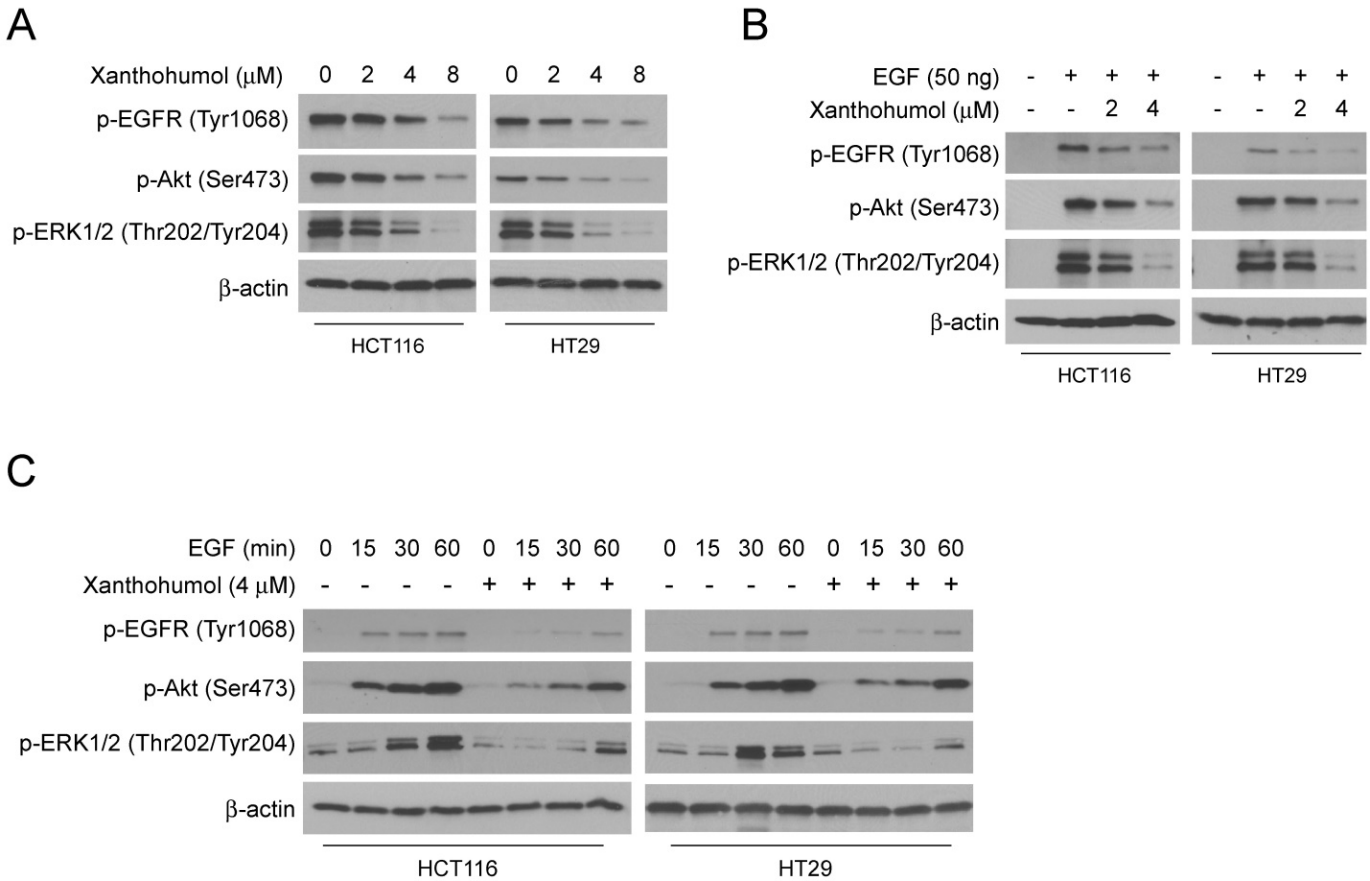
Xanthohumol inhibits EGFR signaling in colorectal cancer cells. A, HCT116 and HT29 cells were treated with different doses of xanthohumol and harvested for IB analysis. B, HCT116 and HT29 cells were serum-starved overnight, then pre-treated with various doses of xanthohumol for 2 h, treated with EGF for 30 min and harvested for IB analysis. C, HCT116 and HT29 cells were serum-starved overnight, then pre-treated with 4 μM xanthohumol for 2 h, and treated with EGF for different time points. Cell lysates were harvested for IB analysis.

**Figure 5:**
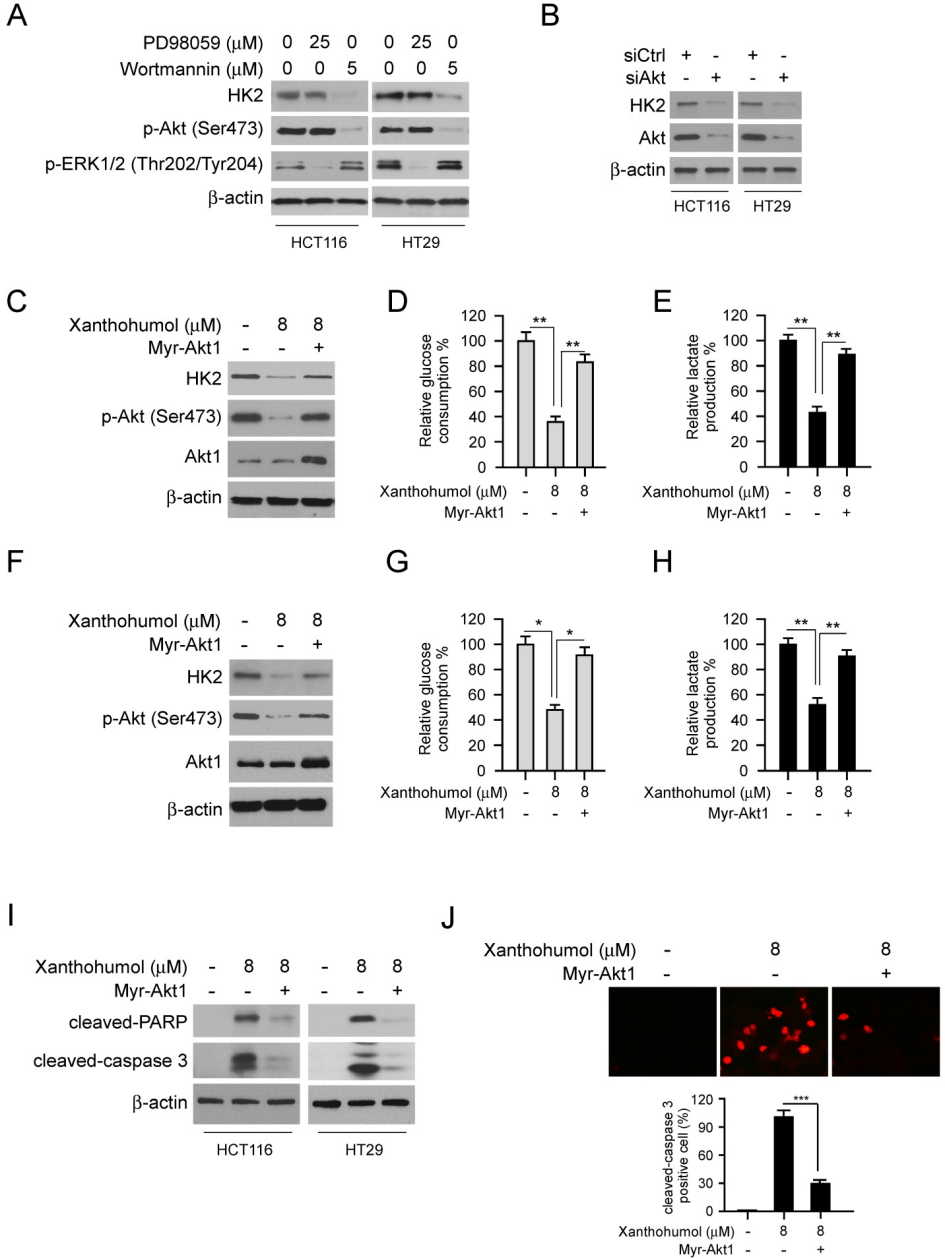
Down-regulation of Akt signaling is required for xanthohumol-mediated glycolysis suppression. A, HCT116 and HT29 cells were treated with PD98059 or wortmannin. Cell lysates were subjected to IB analysis. B, HCT116 and HT29 cells were transfected with siCtrl or siAkt, cell lysates were subjected to IB analysis. C, HCT116 cells were transfected with Myr-Akt1, cell lysates were subjected to IB analysis. D and E, glucose consumption (D) and lactate production (E) in Myr-Akt1 transfected HCT116 cells. F, HT29 cells were transfected with Myr-Akt1, cell lysates were subjected to IB analysis. G and H, glucose consumption (G) and lactate production (H) in Myr-Akt1 transfected HT29 cells. I, HCT116 and HT29 cells were transfected with Myr-Akt1 and treated with xanthohumol, cell lysates were subjected to IB analysis. J, HCT116 cells were transfected with Myr-Akt1 and treated with xanthohumol, cleaved-caspase 3 was analyzed by immunofluorescence. Data are shown as mean values ± S.D. *p<0.05, **p<0.01, ***p<0.001, a significant difference between groups as indicated.

To further explore the mechanism of how xanthohumol inhibits HK2 and glycolysis, we investigated the signaling pathways in xanthohumol-treated CRC cells. Because the epidermal growth factor receptor (EGFR) is recognized as an essential player in CRC initiation and progression, we fist tested EGFR activity with xanthohumol treatment. The results showed that xanthohumol suppressed the phosphorylation of EGFR, and EGFR downstream kinases Akt and ERK1/2 in a dose-dependent manner (Figure 4A). Moreover, pretreated with xanthohumol markedly down-regulated EGF-induced EGFR signaling activation in HCT116 and HT29 cells (Figure 4B). The immunoblotting data further confirmed that xanthohumol delayed the activation of EGFR signaling at various time points (Figure 4C). To determine which downstream kinase is required for xanthohumol-mediated HK2 suppression, HCT116 and HT29 cells were treated with Akt or ERK1/2 inhibitor and subjected to immunoblotting analysis. The result indicated that suppression of Akt, but not ERK1/2, decreased HK2 protein (Figure 5A) and glycolysis significantly (Figure S3A and B). Transient transfection of Akt siRNA induced down-regulation of HK2 protein (Figure 5B), as well as glycolysis consistently (Figure S3C and D). Moreover, overexpression of constitutively active Akt, Myr-Akt1, rescued xanthohumol-induced HK2 suppression (Figure 5C), glucose consumption (Figure 5D), and lactate production (Figure 5E) in HCT116 and HT29 (Figure 5F-5H) cells. The immunoblotting analysis (Figure 5I) and immunofluorescence staining (Figure 5J) results showed that the protein level of cleaved-caspase 3 was decreased in Myr-Akt1 transfected cells, indicating xanthohumol-induced apoptosis was attenuated. These results suggest that xanthohumol-induced HK2 and glycolysis inhibition in CRC cells are partly dependent on the suppression of Akt activation.

### Xanthohumol inhibits tumor growth *in vivo*

**Figure 6:**
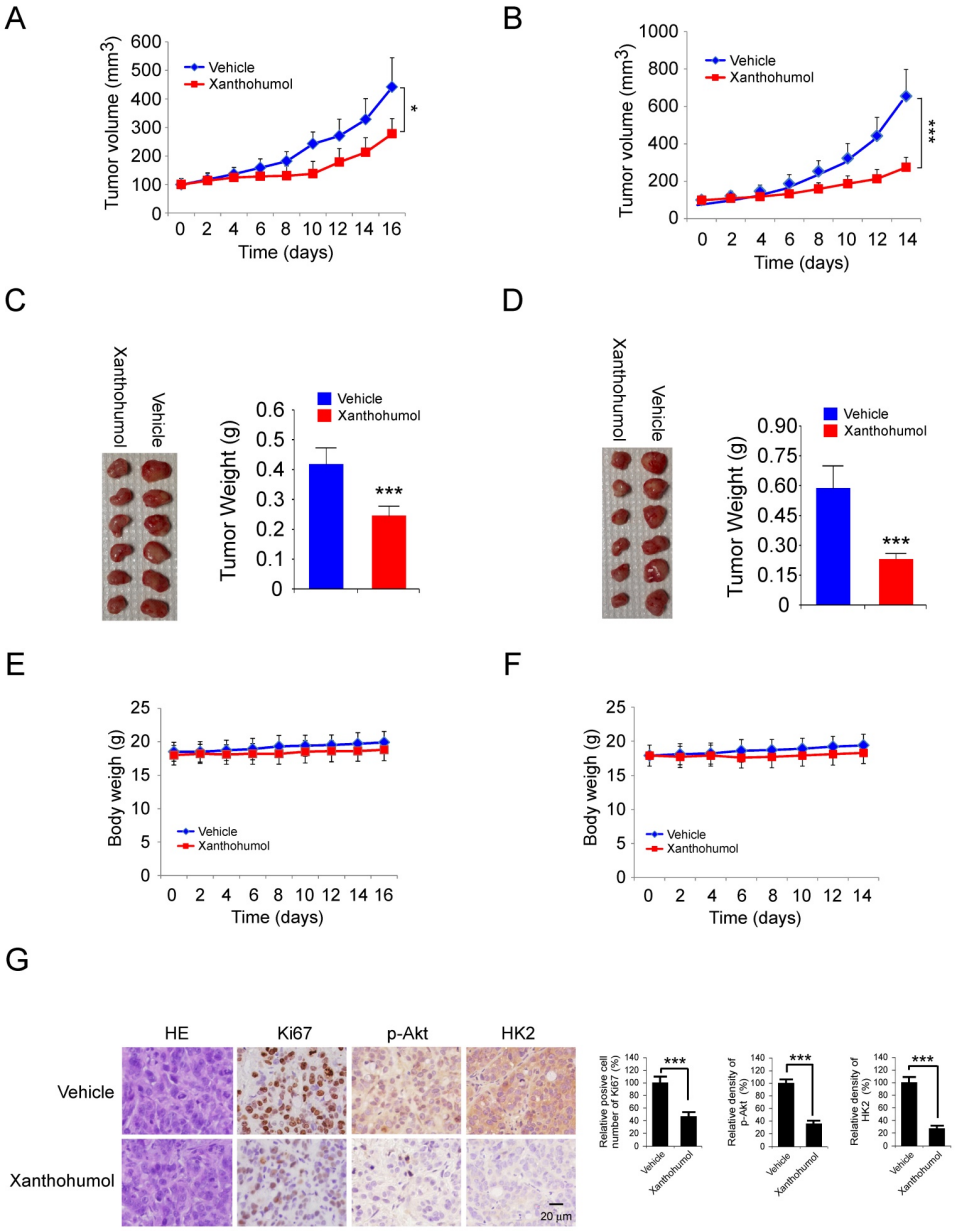
Xanthohumol inhibits tumor growth in colorectal cancer athymic nude mouse model. A and B, tumor volumes of HT29 (A) and HCT116 (B) xenograft tumors treated with xanthohumol or vehicle. C and D, at the treatment endpoint, mice were sacrificed, and HT29 (C) and HCT116 (D) xenograft tumors were removed, weighed, and photographed. E and F, the average body weight of HT29 (E) or HCT116 (F) tumor-bearing mice treated with xanthohumol or vehicle. G. Immunohistochemical examination of Ki67, p-Akt, and HK2 in tumor sections from xanthohumol- or vehicle-treated mice. All panels are of the same magnification. Data are shown as mean values ± S.D. *p<0.05, ***p<0.001, a significant difference between groups as indicated.

We examined the effects of xanthohumol on tumor growth *in vivo*. HT29 and HCT116 cells were injected (s.c.) into the right flank of athymic nude mice. Xanthohumol treatment was initiated when the tumor volume was reached 100 mm^3^. Results indicated that the average tumor size of the vehicle-treated control group was 442 ± 102 mm^3^, whereas the average tumor size of the xanthohumol-treated group was 278 ± 53 mm^3^ (Figure 6A). The similar inhibitory effect was observed in xanthohumol-treated HCT116 xenograft tumors, the tumor volume of the vehicle-treated control group and the xanthohumol-treated group were 655± 143 mm^3^ and 275 ± 61 mm^3^, respectively (Figure 6B). The average tumor weights of the xanthohumol-treated group were significantly smaller than those in the vehicle-treated group in both HT29 (Figure 6C) and HCT116 (Figure 6D) tumor-bearing mice. Moreover, during the treatment period, xanthohumol did not affect body weight significantly (Figure 6E and F). Immunohistochemical staining analysis showed that the positive staining of Ki-67, p-Akt, and HK2 were decreased significantly in the xanthohumol-treated xenografts (Figure 6G). Consistent with the *in vitro* data, these data also indicated that xanthohumol inhibits tumor cell proliferation* in vivo*.

## Discussion

In the present study, we have shown that HK2 is highly expressed in human colorectal cancer tissues and cell lines. Knockout of HK2 reduces tumorigenic properties *in vitro* and *in vivo*. We identified a natural compound, xanthohumol, inhibits the growth of CRC cells and subcutaneous xenograft growth in a nude mouse model. One of the major mechanisms by which xanthohumol exert its effects against CRC seems to be through the suppression of HK2 and glycolysis. Moreover, we demonstrated that downregulation of Akt activity is required for xanthohumol-mediated HK2 and glycolysis inhibition.

Glycolysis can be activated under hypoxic conditions in human cancers. However, the status of glycolysis in CRC cells when cultured under normoxic conditions remains unclear. In the present study, we found that under normoxic conditions, all of the examined CRC cells displayed aerobic glycolysis, as indicated by the increased glucose consumption and lactate production. Hyperactivation of glycolysis supplies a large number of intermediate for macromolecular biosynthesis. Importantly, the accumulation of lactate in tumor tissue resulted in an acidic microenvironment, which is benefited for metastasis and chemotherapy/radiotherapy resistance 16-19. Dysfunction of glycolytic enzymes, such as HK2 and PKM2, is closely correlated with the glucose metabolic reprogramming in human cancer cells. Overexpression of HK2 predicts poor patient survival rate in colon cancer, lung cancer, and glioblastoma 20-23. Importantly, downregulation of glycolytic enzymes and glycolysis suppressed tumor cell growth or induced apoptosis in multiple human cancer types 24-27. Our results showed that knockout of HK2 inhibited the proliferation and glycolysis of CRC cells *in vitro* and *in vivo*, suggesting HK2 a potential target for CRC treatment.

Mitochondria-localized HK2 has been shown to be required for escaping from mitochondrial cell death in several types of human cancer 28-31. Here, we found that xanthohumol suppressed HK2 expression and induced the activation of mitochondrial apoptosis signaling. Our results also suggested that HK2 is essential for xanthohumol correlated metabolic changes and cancer cell apoptosis induction. Overexpression of HK2 significantly attenuated xanthohumol-induced mitochondrial apoptosis and glycolysis suppression.

The present study is the first report to demonstrate that xanthohumol exerts the anti-tumor effect by targeting the aerobic glycolysis in CRC cells.

Recently, numerous natural products have been demonstrated to exert anti-tumor effects via impairment of angiogenesis and metastasis 32-34, inhibition of cell cycle progression 35-37, induction of apoptosis 38, and inhibition of glycolysis 22. Because of the structural diversity and lower side effects, natural compounds have more potential to be applied to tumor chemotherapy in compare with the rational designed small molecules. In the past decades, the beneficial pharmacological properties of xanthohumol were studied systematically, including antioxidant, anti-inflammatory, antibacterial, and anti-tumor activity 10. There were several lines of evidence suggesting that the suppression of kinase activity, downregulation of the transcription factor, and dysfunction of signaling transduction were involved in xanthohumol-mediated tumor suppression 39, 40. However, there has been no study regarding the mechanisms of xanthohumol on the regulation of glycolysis in human CRC cells. In the present study, we found that xanthohumol suppressed HK2 and glycolysis in CRC cells. Moreover, xanthohumol inhibited the activation of the EGFR signaling pathway. Our result indicated that xanthohumol-mediated Akt suppression, but not EGFR downstream kinases ERK1/2, was required for glycolysis inhibition and apoptosis induction.

In summary, our results suggest that the antitumor effects of xanthohumol could be mediated in part by the suppression of glycolysis through direct inhibitory effects on EGFR-Akt signaling and also via induction of apoptosis in tumor cells. Hence, this novel mechanism of xanthohumol makes it a promising agent against CRC cells.

## Supplementary Material

Supplementary figures.
