# Supplementary material for: Xanthohumol inhibits colorectal cancer cells via downregulation of Hexokinases II-mediated glycolysis
Source: Int J Biol Sci. 2019 Sep 7;15(11):2497–508. doi: 10.7150/ijbs.37481 (PMC6775317; doi:10.7150/ijbs.37481)
Supplement: Supplementary file 1 — Supplementary figures. [file ijbsv15p2497s1.pdf]

Supplementary Figure legends

Figure S1. Knockout of HK2 suppresses HT29 xenograft tumor growth. A-C, average tumor volume (A), photographed xenograft tumors (B), and average tumor weight (C) of HT29 sgCtrl and HT29 sgHK2 xenografts. Data are shown as mean values ± S.D. ***$p<0.001$, a significant difference between groups as indicated.

Figure S2. The structure of screened compounds.

Figure S3. Suppression of Akt signaling downregulates glycolysis in colorectal cancer cells. A and B, glucose consumption (A) and lactate production (B) in HCT116 and HT29 cells treated with PD98059 or wortmannin. C and D, glucose consumption (C) and lactate production (D) in HCT116 and HT29 cells transfected with siCtrl or siAkt. **$p<0.01$, ***$p<0.001$, a significant difference between groups as indicated.

Fig. S1

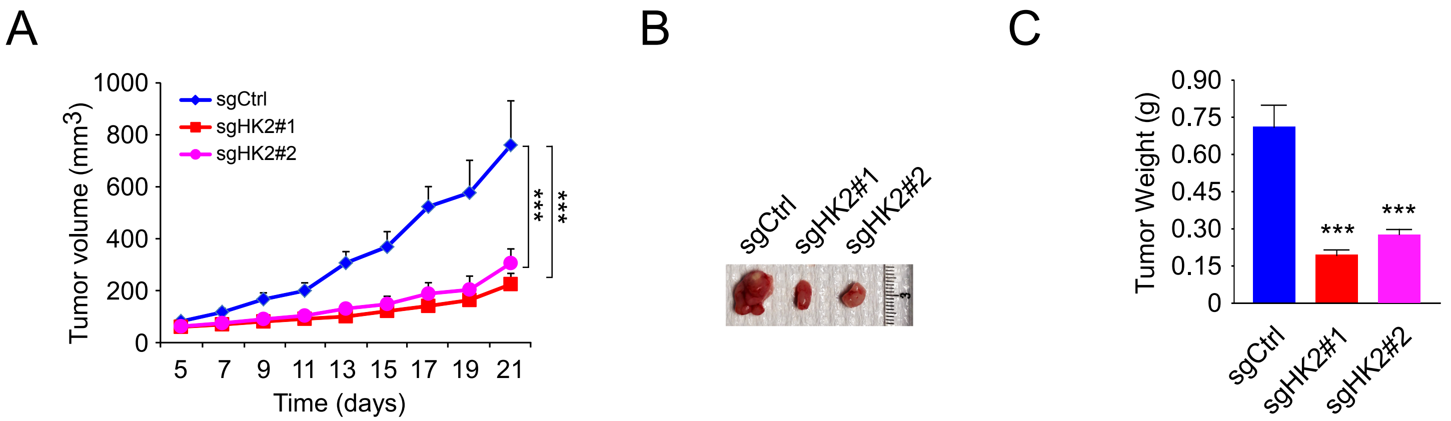

A
B
C
Tumor volume (mm3)
1000
800
600
400
200
0
5
7
9
11
13
15
17
19
21
Time (days)
sgCtrl
sgHK2#1
sgHK2#2
***
***
sgCtrl
sgHK2#1
sgHK2#2
Tumor Weight (g)
0.90
0.75
0.60
0.45
0.30
0.15
0
***
***
sgCtrl
sgHK2#1
sgHK2#2

Fig. S2

Diosmetin

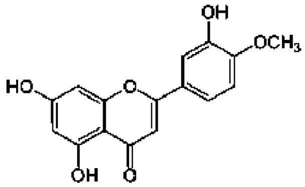

Nobiletin

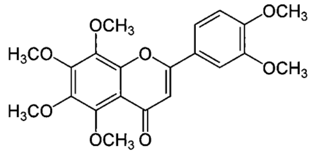

Osthole

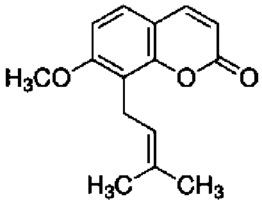

Sinomenine

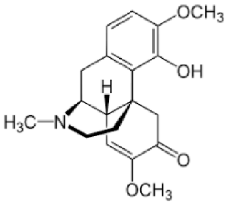

Xanthohumol

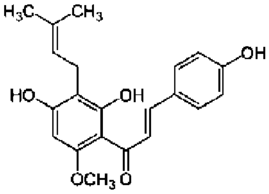

Isoliquiritigenin

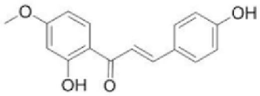

Oxymatrine

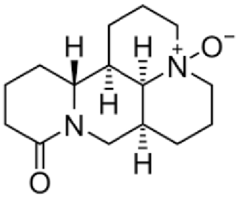

Bergenin

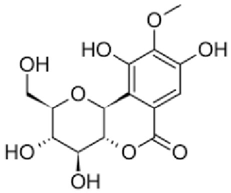

Butein

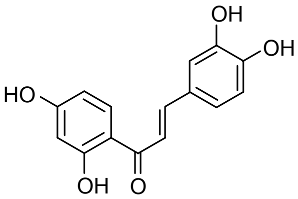

Sophocarpin

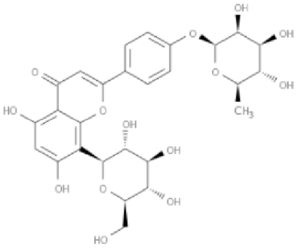

Curcumol

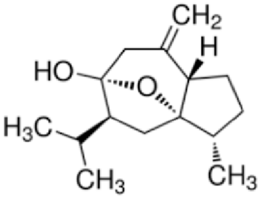

Sesamin

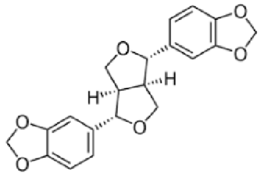

Phlorizin

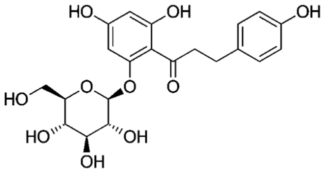

Gastrodin

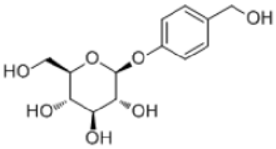

Limonin

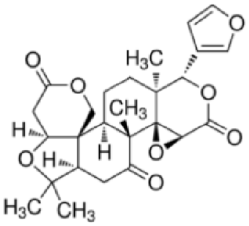

Paeonol

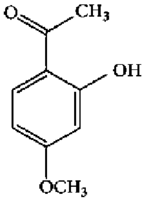

Fig. S3

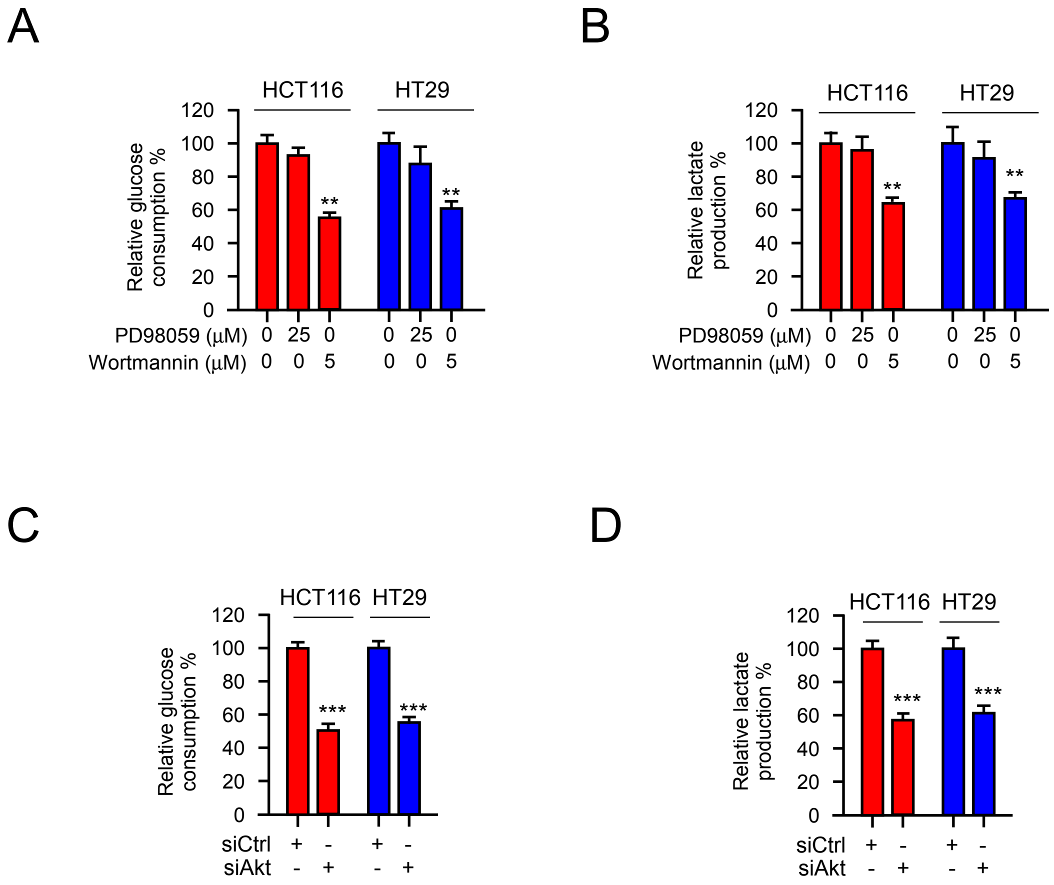
A
HCT116
HT29
Relative glucose consumption %
120
100
80
60
40
20
0
**
**
PD98059 (μM) 0 25 0 0 25 0
Wortmannin (μM) 0 0 5 0 0 5
B
HCT116
HT29
Relative lactate production %
120
100
80
60
40
20
0
**
**
PD98059 (μM) 0 25 0 0 25 0
Wortmannin (μM) 0 0 5 0 0 5
C
HCT116
HT29
Relative glucose consumption %
120
100
80
60
40
20
0
***
***
siCtrl + - + -
siAkt - + - +
D
HCT116
HT29
Relative lactate production %
120
100
80
60
40
20
0
***
***
siCtrl + - + -
siAkt - + - +
